# Evaluation of Four Methods to Determine the Degree of Cure of Melamine-Based Direct Pressed Laminates on Particleboards: Two Improved UV Absorption Methods, the Kiton Test, and Near Infrared Spectroscopy

**DOI:** 10.3390/ma18010117

**Published:** 2024-12-30

**Authors:** Mark Meder, Carsten Mai, Dirk Lukowsky

**Affiliations:** 1 Surfactor Germany GmbH, 38170 Schoeppenstedt, Germany; mark.meder@surfactor.com; 2 Department of Wood Biology and Wood Products, Georg-August-Universität Göttingen, 37077 Gottingen, Germany; cmai@gwdg.de; 3 Fraunhofer WKI, 38108 Braunschweig, Germany

**Keywords:** melamine coating, melamine–formaldehyde, DPL, degree of cure, overcure, wood based panels, NIR, resistance to cracking, hydrolysis, Kiton

## Abstract

Despite its importance, the determination of the degree of cure of melamine-based laminates often relies on tests with limited accuracy and validity. Undercured surfaces may suffer insufficient resistance to scratching and heat as well as substandard surface quality. Overcured melamine surfaces tend to crack and entail the inefficient utilization of the press—the panels could have been pressed for a shorter time. Four methods to determine the degree of cure of a melamine resin coating under industrial conditions were compared: the Kiton test, the most common method in industry, Near Infrared Spectroscopy (NIR) as a modern technique that allows for inline-measurements, and two novel hydrolysis methods. Each test was conducted on the same 18 panels. Each panel differed in its resin system or its degree of cure, which was adjusted by varying the pressing duration and temperature. The four methods tested were all capable of determining the degree of cure to some extent, but their applicability, the delay between the curing of the melamine resin at the final stage of production and the availability of results, and the investment and workload differ greatly. Determining the critical overcure turned out to be the major challenge. Differentiation between slight overcure, which did not affect the cracking resistance, and severe overcure, which produced surfaces with a high tendency to cracking, was possible using the NIR-based method and the two novel hydrolysis methods but not with the widely used Kiton test.

## 1. Introduction

Papers impregnated with melamine and/or urea resins dominate the market for coatings of engineered wood for indoor use [1,2]. Of note, 2.5 billion m^2^ of paper-based surfaces are produced globally each year, with an increasing trend [3]. Typical products are laminates, namely High Pressure Laminate (HPL) and Continuous Pressure Laminate (CPL), as well as thin paper coatings, namely finish foils and Direct Pressed Laminate (DPL). The latter is also known as Melamine Faced Board (MFB), Thermally Fused Melamine (TFM), or simply film-faced panels [4,5,6].

Melamine resins are hard, durable, and resistant to thermal stresses and chemicals except strong acids [7]. The “laminates” referred to herein, HPL, CPL, and DPL, achieve their outstanding surface quality through the final curing of melamine–formaldehyde (MF)- or phenol–formaldehyde (PF)-impregnated papers (called B-stage papers or films) under pressure and heat.

The first curing step of the resin (referred to as conversion from the a-stage to the b-stage) is achieved during the convection drying of the impregnated papers at usually 130 °C to 160 °C. This precuring requires a careful balance between the remaining reactivity and robustness of the impregnated papers during storage [8]

The final cure of the impregnated papers results from hot pressing. In the hot press, the precured resin liquefies at temperatures of commonly 130 °C to 190 °C, and the molecular weight increases to the desired level due to crosslinking via methylene and methylene–ether bridging [8,9]. Müller et al. [10] observed that cross-linking is not finalized during the typical short hot pressing times. A certain part of curing takes place outside the press during the cooling stage. During the curing process, the resin “fuses” inseparably with additional films (HPL and CPL) or directly with the engineered wood (DPL).

The degree of cure is responsible for a multitude of the coating’s properties. Incorrect adjustment of this important production parameter may lead to a variety of defects. Surfaces with an insufficient degree of cure (undercure) suffer from low resistance to chemicals and abrasion. They can easily be stained, the surface may appear cloudy or mushy, air pockets can form right after hot pressing or after contact with hot materials, and the film may even have insufficient adhesion to the other films or the engineered wood. If the cross-linking is allowed to exceed the desired level (overcure), the material may be prone to edge breakouts and cracking. Cracks can form at the edges during formatting or later across the surface. These latter cracks may cause major problems if they occur after the panels have been built into furniture or flooring. The cost of replacing these defective parts can become very high [11]. This is especially unpleasant considering that most of the films that exhibit these cracks account for only a marginal fraction of the material costs. Besides the prevention of these possible shortcomings in the material’s properties, a precise determination of the degree of cure and, in particular, the identification of overcure allows for the optimization of pressing parameters, thereby increasing productivity and saving energy costs.

The kinetics of the curing process of melamine resins were studied in detail with Differential Scanning Calorimetry (DSC) [9,10]. However, for industrial processes, cheaper and, above all, faster methods are required to detect and prevent undercure or overcure as fast as possible. Due to the post-curing behavior of melamine resins, online control of curing is probably less feasible for quality control than the analysis of already pressed laminated samples [10].

An earlier method to determine the degree of cure by quantifying the amount of formaldehyde extracted in boiling sulfuric acid was described by Schröder et al. [12]. This method is rather time consuming. It cannot be used for DPL either due to formaldehyde emission from the inseparably attached and usually formaldehyde-emitting substrate. Guenther [13] showed a method of using shore hardness to determine the degree of cure of melamine–formaldehyde-molding compounds. The relatively soft substrate and the heterogeneous composition due to the paper fibers make this method inadmissible for laminates. Scheepers et al. [14] describe a method to determine the degree of cure of melamine resin using Raman spectroscopy.

Dielectric analysis (DEA) can be regarded as a very promising method for monitoring the cure of thermosetting resins. DEA allows for in situ monitoring (within the hot press) of the curing process. Paper-thin (and cheap) DEA sensors are positioned between the substrate and the impregnated paper. The mobility of ions representing the degree of cure is recorded. The general applicability of DEA for melamine resins was demonstrated by Müller et al. [10]. Gupta et al. [15] demonstrated a good correlation between DEA and Differential Scanning Calorimetry (DSC) in monitoring the cure of phenol–formaldehyde (PF) resins.

The so-called Kiton or acid test is still widely used in industry to test the degree of cure. This method is specified for instance in the Australian/New Zeeland standard [16], but it is used in many variations worldwide. Most companies have implemented their own individual version of the Kiton test. The test involves the application of a mixture of sulfuric or hydrochloric acid and a red dye to the surface. After typically two hours, the mixture is removed and the surface is assessed in terms of stain intensity and change in gloss level. It is assumed that the resulting discoloration is proportional to the degree of resin cure. A surface without any signs of acid attack and staining is accordingly classed as overcured.

In the last decade, NIR spectroscopy has gained interest as a method to determine the degree of cure [17,18]. The patent EP 3238934 A1 [19] describes a possible measurement by using NIR spectra ranging from 700 nm to 2000 nm. The most notable advantage of this method is that it allows for inline measurements in CPL production. However, the method is most likely not suitable for dark and varicolored surfaces.

As an alternative to the visual assessment of the acid test, the amount of melamine hydrolyzed by the acid may be quantified by its UV absorption. Due to its weak UV absorption, hydrochloric acid is particularly suitable for this procedure. The acid is applied to the surface for a predetermined period and then transferred to a UV spectrometer where the UV absorbance at 237 nm is recorded [11]. This principle was used as the basis for the hydrolysis methods examined. Ambient temperature was identified as an impactful influencing factor for these methods [20]. The chemical reactions follow the van’t Hoff rule: an increase in ambient temperature of 10 K can cause the reaction rate to double or even quadruple. The Kiton test, which is also based on these reactions and is therefore influenced in the same way, does not have any specifications regarding the ambient temperature

In this paper, we compare the results derived from several methods and describe advances in the measurement of the degree of cure using novel surface and powder hydrolysis methods. The two hydrolysis methods have been developed to speed up the measurements (crucial in industrial processes) and to overcome the known weaknesses of the commonly used Kiton test.

## 2. Materials and Methods

**Figure 1:**
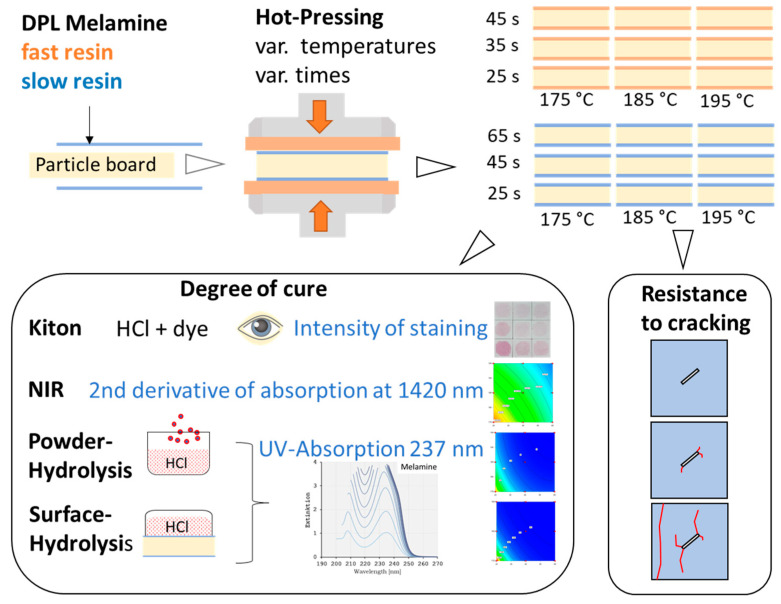
Graphical overview of the methods used: Two sets of melamine-impregnated papers were hot pressed using three pressing times and three temperatures. The degree of cure was tested with four methods. Additionally, the cracking resistance of the cured papers was assessed.

A graphical overview of the methods used is shown in Figure 1.

Film-faced panels with a broad range of degree of cure (from undercured to harsh overcured) were prepared as test materials. In total, 18 boards with nine curing conditions and two different films were used. Each of these 18 reference materials was a 1500 × 1000 × 16 mm particleboard that was film-faced using a Dieffenbacher cycle press (1850 mm × 1250 mm, 15,000 N) at the Fraunhofer WKI. The particleboard used was for non-load-bearing applications for interior use in dry conditions (class 2 EN 312) for DPL application. To achieve the panel’s different degrees of cure, the temperature and duration of hot pressing were adjusted (175 °C, 185 °C, or 195 °C and 25 s, 35 s, 45 s, or 65 s). The pressure was kept at 2.5 MPa for all boards. One film with a slow-curing melamine resin and one film with a fast-curing melamine resin were used. Both films were supplied by an industrial partner.

**Figure 2:**
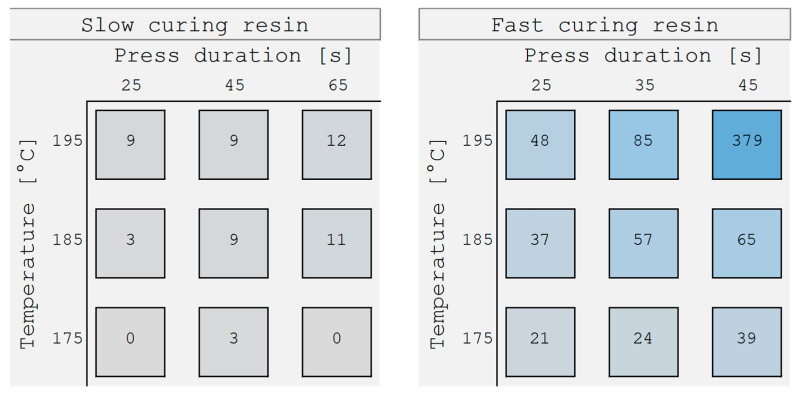
Cracking susceptibility (cumulative crack length in mm) resulting from the different pressing temperatures and durations of the 18 panels examined. The higher the value (also indicated by the increasing blue staining of the background squares), the lower the panel’s resistance to cracking.

Cracking resistance (Figure 2) was determined as cumulated crack length according to the method described by [20]. The method is based on [6] but includes two cracking initials on the 250 mm × 250 mm samples: a 5 mm borehole and a 60 mm long groove. The samples were conditioned at 21 °C, 65% RH, and then stored for 24 h at 70 °C. After 30 min of storage at room temperature, the cumulated crack length (cracking susceptibility) was assessed.

The degree of cure of these 18 coatings was measured with each of the following methods: the industry standard method, called the Kiton test, an NIR-based measurement, and two novel hydrolysis methods, “surface-hydrolysis” and “powder-hydrolysis”.

The Kiton test was performed in accordance with [16]. The surface was exposed to a sulfuric acid/dye solution for 2 h. To avoid evaporation, the acid was covered with flipped watch glass shells. Afterward, the solution was removed, and changes in the color and gloss of the surface were graded. The grades range from rating 1 for faint marking, no surce rise, and no loss in gloss to class 5 for very definite markings or very definite surface rise. According to [18], the amount of discoloration and the change in gloss represent the degree of cure.

To record the NIR spectrum, the surface of each sample was scanned four times (at 0°, 90°, 180°, and 270° rotation of the sample). The data presented are the second derivative of the absorption at 1420 nm. The measurements and calculations were carried out by Perten Instruments GmbH, Germany. We received no information about the instrument used for measurement.

**Figure 3:**
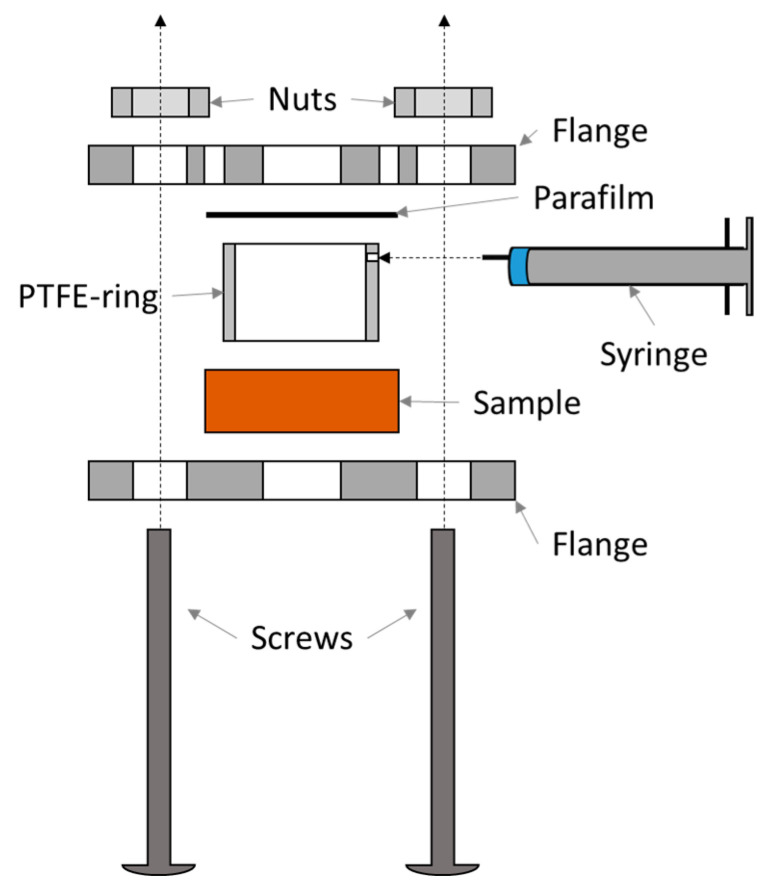
Assembly for surface hydrolysis.

Surface hydrolysis was performed on acetone-cleaned surfaces of a sample (50 mm × 50 mm). A PTFE ring was pressed between the surface of the panel and a sheet of Parafilm using flanges and screws (Figure 3). The apparatus and a syringe filled with 5 mL hydrochloric acid (20%) were stored for 20 min in an oven at 40 °C. Then, the acid in the syringe was injected into the ring through a small hole. After 30 min, the acid was collected, and its extinction was measured at 237 nm in a UV spectrometer (Cary 300 Scan, Varian; 1 cm quartz glass cuvette). An aliquot of 20% hydrochloric acid was used as a blank. The extinction (*E*_237nm_) was converted into the respective mass of hydrolyzed melamine (m_mel_) using Equation (1). Equation (1) was the result of a calibration series with various amounts of melamine solved in hydrochloric acid for said spectrophotometer. The extinction coefficient of 152.7 *l*/*g* may vary for different spectrometers.

(1) $$m_{mel}=\frac{E_{237nm}}{152.7\;l/g}*5\;mL$$

For novel powder hydrolysis, the sample’s surface was cleaned with acetone before a powder was manually ground off using sandpaper (grain P500). The powder (0.15 mg) was filled into a vial and stored in a water bath at 40 °C. After the vial’s temperature had equalized, 5 mL of pre-heated acid (15% hydrochloric acid at 40 °C) was injected into the vial. After 5 min of incubation, the acid solution was drawn up with a syringe and filtered through a syringe filter with a 0.2 µm pore size. The amount of hydrolyzed melamine was measured and calculated using the same UV spectrometer technique as for surface hydrolysis. The data were analyzed using Design Expert 10.0.1.

## 3. Results

### 3.1. Kiton Test

**Figure 4:**
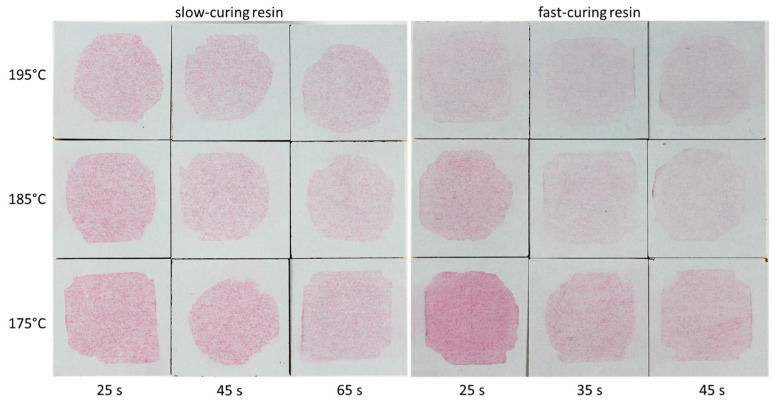
Samples with slow-curing (**left**) and fast-curing resin (**right**) after the Kiton test. The shapes of the stained areas differ because round as well as rectangle watch glasses were used.

The surface discoloration of the Kiton test specimens more or less corresponded to the pressing regime of the samples, with light discoloration for overcured samples and severe discoloration for undercured samples (Figure 4).

**Figure 5:**
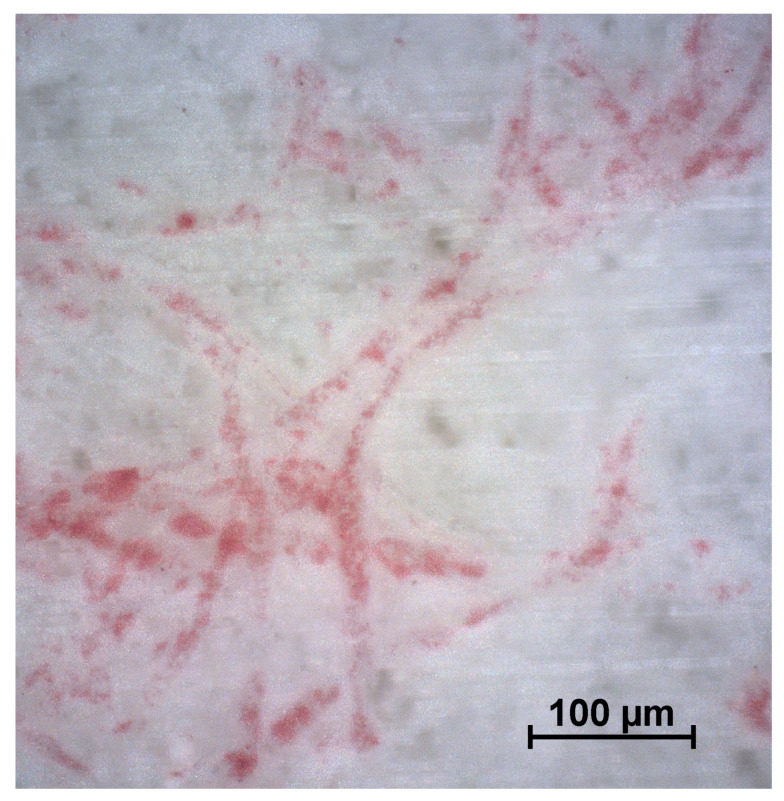
Reflected light microscopic images of a melamine surface after the Kiton test. The red longish threads are stained near-surface fibers.

The observed change in surface color can lead to serious misinterpretation where paper fibers are present near the surface [11]. The acid–dye solution can dissolve thin resin overlays and expose adjacent fibers. Such paper fibers can then act as a wick, absorbing large quantities of the acid–dye solution. Discoloration due to dye absorption by the resin or staining of the paper fibers cannot be distinguished visually without adequate magnification. This wick effect was found in the samples examined. Stained longish markings can be seen in the microscopic images (Figure 5). These stains originated from the described near-surface fibers being primarily discolored, while areas without near-surface fibers present showed hardly any discoloration.

The Kiton test differentiated between highly overcured and highly undercured samples. On the other hand, differences relevant to practical performance—namely cracking resistance—were not represented by the Kiton test. For example, the discoloration of the 25 s/195 °C and 45 s/195 °C pressed fast-curing samples was similar in the Kiton test, but the cracking susceptibility of these two samples was very different (48 mm versus 379 mm).

### 3.2. NIR

**Figure 6:**
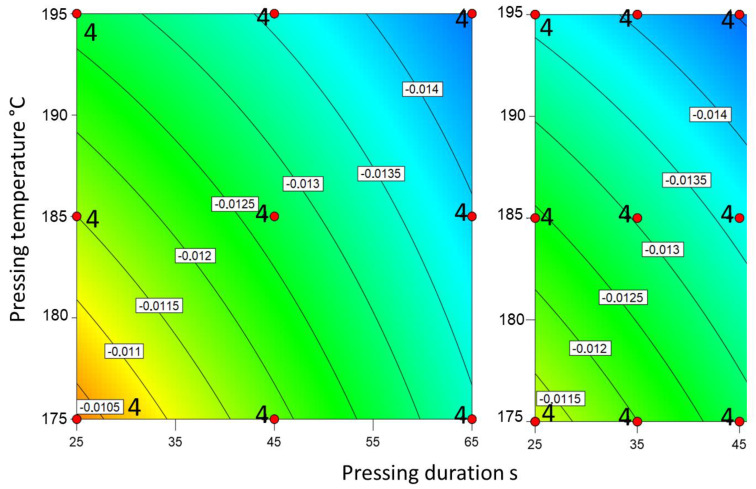
NIR absorption values at 1420 nm for the slow-curing resin (**left**) and the fast-curing resin (**right**). The red dots mark the production parameters of the boards. The numbers next to the dots represent the number of tests performed with said board. The scales of the axes are kept constant. Therefore, the graph for the fast-curing resin is narrower than the graph for the slow-curing resin.

The NIR data showed a good representation of the degree of cure as expected from the pressing conditions (Figure 6). Undercured samples exhibited low absorption; overcured samples exhibited high absorption. The dependency is seemingly linear with a similar resolution for overcured and undercured samples.

Patent EP 3238934 A1 [19] describes a similar method based on NIR measurements to quantify the degree of cure. Instead of a single wavelength (here 1420 nm), the patented method examines an NIR spectrum (500 nm to 2500 nm).

### 3.3. Hydrolysis Methods

**Figure 7:**
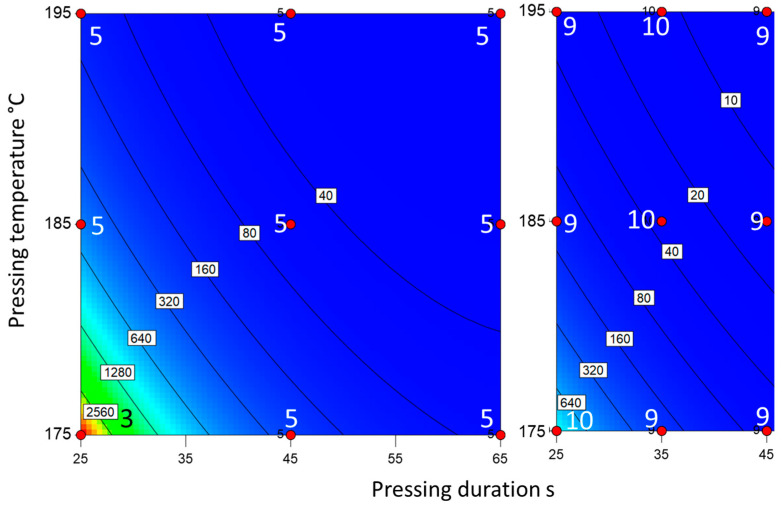
Mass of hydrolyzed melamine [µg] (via surface hydrolysis) for the slow-curing resin (**left**) and the fast-curing resin (**right**). The red dots represent the production parameters of the boards. The numbers next to the dots represent the number of tests performed with said board.

**Figure 8:**
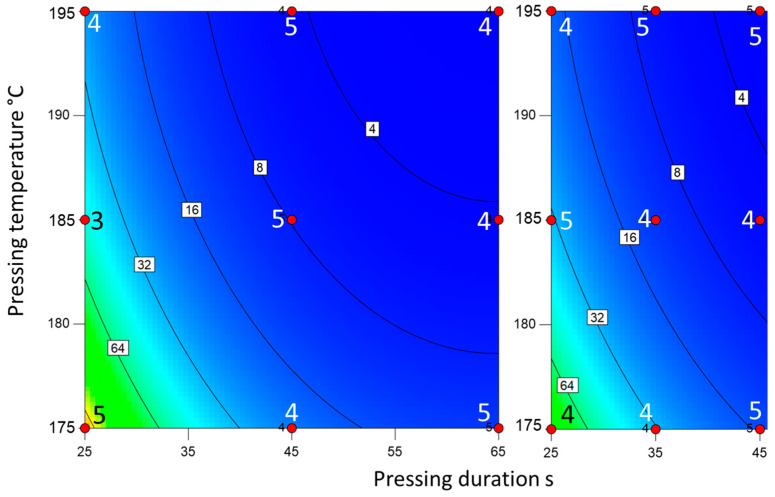
Mass of hydrolyzed melamine [µg] (via powder hydrolysis) for the slow-curing resin (**left**) and the fast-curing resin (**right**). The red dots mark the production parameters of the boards. The numbers next to the dots represent the number of tests performed with said board.

The novel surface hydrolysis and the newly developed powder hydrolysis methods showed a similar representation of the degree of cure as expected from the pressing conditions (Figure 7 and Figure 8). The measured values for powder hydrolysis were lower than those for surface hydrolysis. However, the degree of cure was still distinguishable due to the spectrophotometer’s high resolution. The relationship between the degree of cure and the amount of hydrolyzable melamine is logarithmic for both methods. Therefore, the measured values for slight overcure and severe overcure differed only slightly, but the differences were still within the method’s resolution. Unlike the Kiton test, both hydrolysis methods were able to identify those panels with potentially problematic cracking resistance due to severe overcure.

A disadvantage of powder hydrolysis is the high workload and the required high accuracy regarding hydrolysis time and temperature in comparison to the Kiton test. In the Kiton test, an acid–dye solution (which can be mixed in advance for multiple tests) is applied to the surface. After two hours the discoloration is assessed. In between, the auditor can pursue other assignments. During powder hydrolysis, the powder is ground off the surface, weighed, hydrolyzed with acid, and filtered off, and the amount of melamine is quantified using a UV spectrometer. While the hydrolysis methods have a higher workload, their results have higher validity.

All compared methods were capable of determining the degree of cure to some extent, but their industrial applicability, the delay between production and the availability of results, as well as the required investment and workload, differ greatly. 

The Kiton test is the simplest of all of the methods compared in this study. The workload is minimal, as the auditor only needs to apply the solution and check for discoloration two hours later. However, this test is highly influenced by ambient temperature, even though no predefined specification for this parameter exists. In contrast, the hydrolysis methods use a predefined test temperature. Pigments and colors in the resin can interact with the Kiton test solution, leading to inconclusive results. Additionally, the dye in the Kiton test can be absorbed by near-surface fibers, causing the resulting discoloration to be misinterpreted as undercure. 

NIR proved to be a reliable method for determining the degree of cure of melamine resins, with a resolution for overcured and undercured samples similar to that of the hydrolysis methods. As NIR can be carried out in-line with relatively low labor costs, the investment might be worthwhile. However, the NIR method requires another method for calibration, and dark colors distort the NIR signal, making measurements unreliable. 

The hydrolysis methods provide improved resolution of the degree of cure since they use an objectively measured value (UV extinction) instead of a subjectively assigned degree of cure. The powder hydrolysis method takes five minutes, making it significantly quicker than the two-hour Kiton test. However, hydrolysis methods involve a comparable workload, either grinding the powder and weighing portions or cutting the panel into small samples that fit the assembly (Figure 3). 

It is important to mention that it is not possible to specify universally valid thresholds for undercured or overcured samples with any of the compared methods. As with any known method for determining the degree of cure, the value for an optimally cured surface must be established individually for each melamine coating product. It can be assumed that the resin composition (molar melamine-to-formaldehyde ratio, hardener type, and hardener concentration) is a major influencing factor. If this hypothesis proves to be true, manufacturers will not need to specify thresholds for each product individually but only for product groups that use the same resin-hardener system. 

## 4. Conclusions

The two novel hydrolysis methods presented in this study have proven to be reliable methods for measuring the degree of cure of melamine–formaldehyde coatings. Depending on the use case, each of the four compared methods has advantages and disadvantages for industrial use (Table 1). However, only the two novel hydrolysis methods and NIR are capable of distinguishing between a highly cured panel with still tolerable cracking resistance and a further cured panel with problematic cracking resistance. Despite its widespread use in industry, the Kiton test presents significant drawbacks regarding accuracy, reproducibility, and the delay between production and result availability.

## Figures and Tables

**Table 1:** Advantages and disadvantages of the four methods including an estimation of the workload. The resolution of undercure and overcure expresses the ability of the method to differentiate between moderate, high, and extreme overcure/undercure.

Parameter	Kiton	NIR	Powder Hydrolysis	Surface Hydrolysis
Estimated Workload	1 min	online	10 min	5 min
Estimated Time until Results	2 h	seconds	10 min	35 min
Nature of Results	subjective	objective	objective	objective
Complexity of the Procedure	simple	simple/medium *	medium	simple
Risk of Misinterpretation	high	medium	low	low
Undercure Resolution	medium	high	High	high
Overcure Resolution	none	medium/low	medium/low	medium/low
Financial Investment	low	high	medium	medium

* Depending on the calibration method.

## Data Availability

The original contributions presented in the study are included in the article, further inquiries can be directed to the corresponding author.
